# Allergic reactions during general anaesthesia in Slovenian children

**DOI:** 10.1186/2045-7022-4-S3-P142

**Published:** 2014-07-18

**Authors:** Tina Vesel, Anja Koren Jeverica, Mira Šilar, Alojz Ihan, Jasmina Livk, Mitja Košnik, Peter Korošec, Tadej Avèin

**Affiliations:** 1 University Children's Hospital, University Medical Center, Department of Allergology, Rheumatology and Clinic, Slovenia; 2 University Clinic of Respiratory and Allergic Diseases, Laboratory for Clinical Immunology and Molecular g, Slovenia; 3 Medical faculty, University of Ljubljana, Ljubljana, Institut of Microbiology and Immunology, Slovenia; 4 University Clinic of Respiratory and Allergic Diseases, Department of Allergology, Slovenia

## Introduction

The data on incidence and causes of anaphylaxis during anesthesia in children are lacking. Latex, neuromuscular blocking agents (NMBAs) and antibiotics are most frequently described causes but more data including assessment of cross reactivity of drugs and information of subsequent anaesthesia would be useful.

## Methods

We prospectively included 13 children (8 girls, aged 2 to 13 years) that were referred to our department for investigation the cause of systemic allergic reaction during anaesthesia between 2010-13. Diagnostic procedure included detailed history, skin prick and intradermal testing, detection of specific IgE antibodies (to suxametonium, morphin, latex, beta-lactams (BL), chlorhexidine) and provocation tests (nonsteroidal anti-inflammatory drugs, benzodiazepines and latex). We performed also basophil activation tests (BAT) with BL, NMBAs, midazolam, propofol and glucocorticoids. Stimulation index (SI) >2 was considered positive.

## Results

Five children presented with anaphylaxis and eight with urticaria/angioedema. Seven of them had reaction during their first anaesthesia. Seven children had positive testing results to one group of drugs or one substance (two to NMBAs, two to BL, one to glucocorticoids, one to opioids and one to latex). Three children presented with positive testing results to more than one group of drugs (one to NMBAs, opioids and benzodiazepines, one to NMBA, benzodiazepines and isopropyl alcohol and one to benzodiazepines and garamicin). Two children had dermographism. In one child parents did not want to start diagnosing procedure. Cross- reactivity within the group of medication was found in eleven children (among NMBA, BL, glucocorticoids, benzodiazepines and opioids). BAT for NMBA was positive in three of four children allergic to NMBAs, in both children allergic to BL, in one child allergic to glucocorticoids and in one of three children allergic to midazolam. Four children had uncomplicated following anaesthesia, one girl vomited during the next anaesthesia.

## Conclusions

Causes of anaphylaxis during anaesthesia in children are diverse. Multiple positive testing results complicate further potential anaesthesia. BAT can be a useful additional diagnosing method in immediate hypersensitivity reactions occurring during anaesthesia.

